# Starving for Data: Eating Disorders Prevalence and Research Gaps in Southern Africa

**DOI:** 10.1002/eat.70021

**Published:** 2026-01-07

**Authors:** Bernou Melisse, Jojanneke M. Bijsterbosch, Lot Sternheim

**Affiliations:** ^1^ American Center for Psychiatry and Neurology Abu Dhabi Abu Dhabi UAE; ^2^ Co‐Eur Utrecht the Netherlands; ^3^ Department of Clinical Psychology Utrecht University Utrecht the Netherlands; ^4^ Department of Medical and Clinical Psychology Tilburg University Tilburg the Netherlands

**Keywords:** anorexia nervosa, binge‐eating disorder, bulimia nervosa, disordered eating, eating disorders, eating disorders symptoms, epidemiology, prevalence, Southern Africa

## Abstract

**Objective:**

Studies reporting prevalence estimates of eating disorders in Southern Africa are scarce. To fill this gap, the present Research Forum reviews existing literature on the prevalence of eating disorders, including as assessed by clinical interviews, screeners, and self‐reported behaviors, among individuals in Southern Africa. Key recommendations for future research directions are provided.

**Method:**

The present Research Forum was registered in the International Prospective Register of Systematic Reviews (#541032). Study selection followed the Preferred Reporting Items for Systematic Reviews and Meta‐Analyses guidelines. Peer‐reviewed studies in all languages were searched across multiple databases up to March 2025 using predefined keywords; 209 studies were screened for content.

**Results:**

A total of 16 English studies in six countries were included, all published between 1981 and 2025. Eating disorder point prevalence, defined as the proportion of clinically diagnosed cases, was 0.7%. Named eating disorder prevalences ranged between 0.5% and 4.7%, and high prevalences of pica were found. Prevalence based on screening instruments, indicating increased risk (i.e., proportion scoring above the clinical cutoff) ranged from 3.5% to 37.5%.

**Discussion:**

Studies were few, methodologically diverse, and culturally heterogeneous. Key recommendations for future research include examining factors related to globalization and food insecurity and their potential interplay with the manifestation of eating disorder symptomatology, exploring the roles of ethnicity and sex, evaluating the cultural appropriateness of (Western) standardized assessment tools, and incorporating more diverse samples.

## Summary

- Eating disorder prevalence in Southern Africa ranged from 0.7% to 4.7%.
- Future work should explore how globalization and food insecurity interact with eating disorder symptoms, considering ethnicity and sex.
- Future studies should evaluate the appropriateness of (Western) standardized eating disorder assessment tools and include more diverse samples.

## 1 Background

Although there are strong indications that eating disorders (EDs) are prevalent in Southern Africa (Qian et al. 2021), EDs research remains limited (van Hoeken et al. 2016). There is a large knowledge gap regarding the prevalence estimates of ED in this region (Philip et al. 2023). The present Research Forum directly addresses this gap by synthesizing available literature focused on ED prevalences in Southern Africa. Mapping out the prevalence of EDs in Southern Africa, which includes 16 countries (SADC 1980b, 2024), is both warranted and timely. Importantly, Southern Africa is experiencing increased globalization alongside persistent food insecurity, both of which are associated with ED pathology in other regions (Hazzard et al. 2021; Melisse et al. 2024), yet ED prevalence in Southern African countries is severely understudied and consequently underreported. This underscores the urgent need for prevalence‐focused research in Southern Africa.

Globalization introduces non‐Western regions to Western values and beauty ideals (Carter 2022; Melisse et al. 2020; Salant and Lauderdale 2003) and is associated with a shift from curvy to thin body preferences and thin‐ideal internalization (Gerbasi et al. 2014; Gordon 2001; Nasser et al. 2001). Thin‐ideal internalization is associated with body dissatisfaction (Melisse et al. 2024) and ED pathology (Gerbasi et al. 2014; Swami et al. 2010). In parallel, globalization coincides with a more sedentary lifestyle and increased access to processed foods (Gorrell et al. 2019; Melisse, Fakhri, et al. 2025). These drive both a rise of obesity (Eladawi et al. 2018) and elevated focus on shape and weight as determinants of self‐worth (Melisse and Dingemans 2024). Studies show that EDs do occur in non‐Western regions, especially in those with growing globalization (Melisse et al. 2024).

Food insecurity, defined as a limited access to sufficient, safe, and nutritious food, is especially pronounced in regions with widespread poverty (Pondie et al. 2023), and affects millions in Southern Africa (WorldBank 2024). Food insecurity is associated with ED behaviors, bulimia nervosa (BN), and binge‐eating disorder (BED; Becker et al. 2019; Becker et al. 2017; Hazzard et al. 2021; Hazzard et al. 2020). Early research like the Minnesota Starvation Experiment shows that starvation is associated with cognitive, emotional, and behavioral changes, such as binge eating (Keys 1950). In Western regions, food insecurity is positively associated with feeding and ED pathology (Becker et al. 2019).

Taken together, existing reviews on EDs addressed Western (Hoek 2016; Smink et al. 2016) or broader non‐Western populations (Hernández et al. 2022; Thomas et al. 2016). Only two reviews include EDs in Africa (Philip et al. 2023; van Hoeken et al. 2016), underscoring a notable research gap in EDs research in this region. As highlighted by Philip et al. (2023), addressing the epidemiology of EDs in Southern Africa is both necessary and timely. Specifically, in Southern Africa, globalization is occurring unevenly: Western beauty and body ideals reach young individuals rapidly through social media platforms (e.g., TikTok and Instagram), while local systems such as health education, mental health services, and food insecurity evolve at a much slower pace (Hart and Norris 2024). This imbalance may create a unique vulnerability profile for the onset of EDs and may leave the region unprepared.

The primary aim of the present Research Forum is to present prevalence estimates of EDs, estimates of individuals scoring above a clinical cutoff on a self‐report instrument, and self‐reported ED behaviors or symptoms, while critically reflecting on measurement challenges and the cultural context that may differ from the Western context on how EDs are understood and reported. To advance this under‐studied but important area of ED research in this region, the secondary aim of the present Research Forum is to provide a foundation for future studies that will expand ED understanding and inform care for EDs in Southern Africa. Increased knowledge around prevalence estimates is furthermore essential to improve awareness and access to care.

## 2 Method

### 2.1 Population

The examined population included individuals residing in Southern Africa, specifically in the 16 countries of the Southern African Development Community (SADC; Angola, Botswana, Comoros, Democratic Republic of Congo, Eswatini, Lesotho, Madagascar, Malawi, Mauritius, Mozambique, Namibia, Seychelles, South Africa, United Republic of Tanzania, Zambia, and Zimbabwe) (SADC 1980a), populations who are affected by food insecurity and globalization, among other factors. Comparisons were made between Southern Africa and Western and broader non‐Western regions.

### 2.2 Search Strategy and Study Selection

**FIGURE 1:**
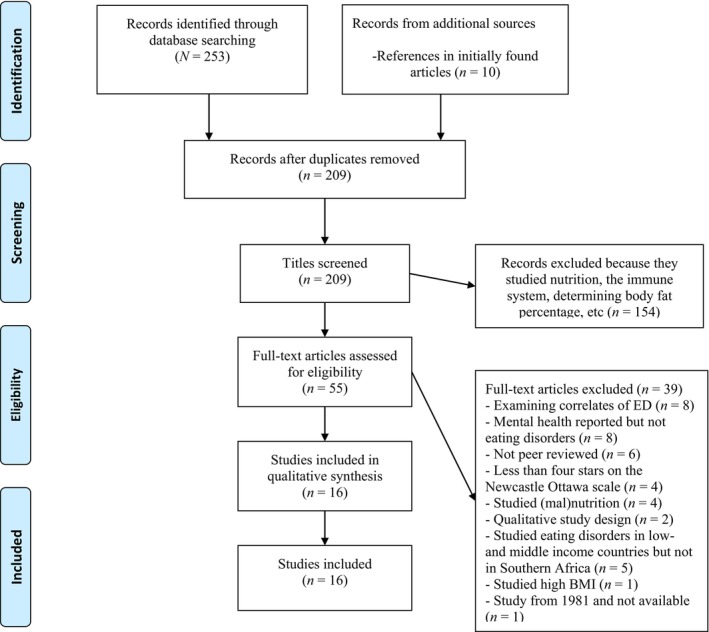
Flow chart of study selection process based on PRISMA.

Methods for systematic reviews were used. The present paper was registered in the International Prospective Register of Systematic Reviews (PROSPERO; registration number: 541032). In addition, study selection was in accordance with the Preferred Reporting Items for Systematic Reviews and Meta‐Analyses (PRISMA) guidelines (Moher et al. 2010). Peer‐reviewed studies published in English and the respective Southern African languages were included, regardless of study design, in order to cover all relevant available data to date. Literature was systematically searched and reviewed from Web of Science, PubMed (Medline), PsycINFO, AfricaBib, and Google Scholar from the earliest available listing (June 1981) up to March 2025. The search aimed to identify studies that reported on EDs, individuals scoring above a clinical cutoff on an ED self‐report, and ED behaviors in Southern African countries. To facilitate the identification of studies, a list of keywords and phrases including a combination of various EDs (e.g., BED), behaviors (e.g., binge‐eating behavior), and countries (e.g., Botswana) was used in English (see Appendix A for the full list of English search terms), and respectively, in the Southern African languages (see Appendix B for the corresponding terms in Southern African languages). Figure 1 shows that the search led to a total of *N* = 253 studies. Moreover, to identify additional suitable studies, reference lists of these publications were searched manually, screening for studies in the relevant region that report about ED and ED behaviors, which resulted in *n* = 10 added studies. Subsequently, identified studies were imported into Endnote, and after deduplication, *n* = 209 studies remained to screen the content. The first author performed a content assessment based on study titles, whereby abstracts and key words were screened for eligibility. The second and third authors performed an additional search. A total of 16 studies were eligible, of which most were conducted in South Africa (*n* = 8). In each of the following countries, a further two studies were conducted in the United Republic of Tanzania, Uganda, and Zimbabwe. In each of the following countries only one study was conducted: Madagascar and Mozambique. No studies were identified in Botswana, Comoros, the DRC, Eswatini, Lesotho, Malawi, Mauritius, Namibia, or Zambia.

### 2.3 Inclusion and Exclusion Criteria

Studies had to meet the following criteria for inclusion: (1) they had to be conducted in, and include residents of one of the SADC countries, and (2) they had to report (e.g., point/annual/12 months/period/lifetime) prevalence of (i) EDs (an ED diagnosis as established through a clinical interview), (ii) prevalence of individuals scoring above a clinical cutoff on a self‐report assessment, indicating that no formal diagnosis was established but being at high risk for an ED, or (iii) self‐reported ED behaviors (e.g., binge eating or purging behavior) or symptoms (e.g., body dissatisfaction). Studies were excluded when they (1) reported about non‐Southern African countries, (2) solely included high body mass index (BMI; kg/m^2^) without ED symptoms, and (3) reported only qualitative data without quantitative data. No restrictions on age or sex were applied due to the limited availability of relevant studies, allowing for a broader inclusion of available data.

### 2.4 Quality Assessment, Publication Bias and Data Extraction

The quality of the included studies was assessed by the Newcastle‐Ottawa scale (Modesti et al. 2016), a seven‐item scale that investigates power, research design, sample, recruitment, and statistical analysis (See Appendix C for the Newcastle‐Ottawa scale), where studies could obtain a maximum score of 10 stars. In accordance with another review conducted in a region dealing with a lack of ED studies (Melisse et al. 2024), studies with at least four stars were included in the present review. Data extraction and the risk of bias assessments were completed by the first author. The data extraction process involved systematically reviewing each included study to extract key variables and study characteristics. Specifically, the following information was extracted: country of study, authors and year of publication, participant details (including sample size, age, and sex), study design (e.g., cross‐sectional, case–control, validation study), and the specific measures used for assessment (e.g., interview data for diagnosis and self‐reports to identify individuals scoring above a clinical cutoff). Finally, the PRISMA guidelines were followed (Figure 1; Moher et al. 2010).

## 3 Results

### 3.1 Study Selection and Characteristics of Studies

Only studies published in English were found. Table 1 shows that *N* = 16 studies met the inclusion criteria, of which *n* = 13 were of a cross‐sectional nature, two validation studies, and one case report. None of the identified studies included longitudinal data. Four studies used interview assessments, including the MINI‐international neuropsychiatric interview (Sheehan et al. 1998), the Structured Clinical Interview DSM (SCID; First et al. 2016), an unspecified clinical interview, and a national health survey. A total of eleven studies included self‐reports such as the Eating Attitude Test‐40 (EAT‐40; *n* = 2; Garner and Garfinkel 1979), Eating Attitude Test‐26 (EAT; *n* = 4; Garner et al. 1982), the Eating Disorder Inventory (EDI; *n* = 2; Garner 1991), the Bulimic Investigatory Test, Edinburough (BITE; *n* = 2; Henderson and Freeman 1987), and the Sick, Control, One stone, Food, Fat (SCOFF; *n* = 2; Morgan et al. 2000). Self‐reports to measure body dissatisfaction were the Body Shape Questionnaire (BSQ; *n* = 1; Cooper et al. 1987), and the Weight Concerns Scale (WCS; *n* = 1; Dias, Maroco, et al. 2015). The administered self‐reports were not described in one study (Ballot et al. 1981). The EAT had good psychometric properties in South Africa (Edwards et al. 2003) and in Tanzania (Eddy et al. 2007), and the WCS was validated in Mozambique (Da Silva et al. 2017). The other questionnaires were not validated for use in Southern Africa. Finally, the majority of studies included in the present Research Forum reported on sex (male/female), whereas gender was not assessed as a multidimensional construct. Accordingly, the analyses are based on sex, as reported in the original studies.

**TABLE 1:** Summary of studies reporting the prevalence of eating disorders, scoring above the clinical cutoff on an eating disorder self‐report assessment, and eating disorder symptoms in Southern Africa.

Country	Author (year)	Local researchers	Study design	Participants (age, sex, ethnicity, socio‐economic status)	Assessment tools	Prevalence
Studies on eating disorder prevalence and individuals scoring above a clinical cutoff on a self‐report instrument						
Madagascar	(Golden et al. 2012)	Yes	CS	Community sample (*N* = 760) Age: > 5 years Sex: females and males Ethnicity: 48.6% Betsimisaraka 51.5% Tsimihety Socio‐economic status: NR	Interview	Prevalence of pica related substances during the prior year geophagy = 53.4%, amylophagia = 85.2%, other pica substances (e.g., charcoal, chalk) = 19.0%. Frequency of pica behaviors was not associated with pregnancy.
Mozambique	(Dias, da Silva, et al. 2015)	Yes	VS	College students (*N* = 185) Age: 18–35 years Sex: female Ethnicity: Mozambican Socio‐economic status: NR	WCS, BSQ	Scored above cutoff WCS (≥ 52; Dias, Maroco, et al. 2015) = 15.7%.
South Africa	(Ballot et al. 1981)	Yes	CS	School students (*N* = 1246) Age unknown Sex: female Ethnicity: NR Socio‐economic status: NR	NR	Suggested point prevalence AN = 2.9% in the past 12 months; based on measured body dimensions and self‐reported body image/eating behaviors, not DSM criteria. This reflects “AN‐like” pathology, not a formal DSM diagnosis.
South Africa	(Le Grange et al. 2006)	Yes	CS	High school and university students (*N* = 895) Age*: M* = 17.2 (SD = 1.7) Sex: female and male Ethnicity: 14.1% African, 57.5% Caucasian, 28.4% Mixed Socio‐economic status: NR	EAT‐26, BITE	Scored above cutoff EAT (≥ 20) and BITE (≥ 25): 3.5%.
South Africa	(le Grange et al. 1998)	Yes	CS	University students (*N* = 1435) Age: 17–25 years Sex: female and male Ethnicity: 28% African, 10% Asian, 52% Caucasian, 10% Mixed Socio‐economic status: NR	EAT‐40, BITE	Scored above cutoff EAT (≥ 30): 9%. Scored above cutoff BITE (≥ 25): 5%.
South Africa	(Szabo and Hollands 1995)	Yes	CS	High school students (*N* = 213) Age: NR Sex: females Ethnicity: NR Socio‐economic status: NR	EAT‐26	Scored above clinical cutoff EAT (≥ 20): African ethnicity = 37.5% Caucasian ethnicity = 20.7%.
South Africa	(Szabo and Allwood 2004b)	Yes	CS	High school students in rural area (*N* = 361) Age 17.87 (SD = 2.77) Sex: females Ethnicity: African (100% Zulu) Socio‐economic status: through paternal occupation. 56% of fathers held unskilled occupations. 2.7% of fathers held professional occupations	EAT‐26	Scored above clinical cutoff EAT (≥ 20): 3.1%
South Africa	(Szabo and Allwood 2004a)	Yes	VS	High school students in urban area (*N* = 1353) Age: NR Sex: females Ethnicity: 43% African, 37% Caucasian, 20% Other Social‐economic status: NR	EAT‐26	Scored above clinical cutoff EAT (≥ 20): 18.7% for students of African ethnicity 18.6% for students of Caucasian ethnicity (other not reported).
South Africa	(Swartz and Sheward 1995)	Unknown	CS	University students (*N =* 1500) Age: < 26 years Sex: female and male Ethnicity: NR Socio‐economic status: NR	NR	Percentage of individuals believing they had eating disorder pathology in the past: African = 15% Caucasian = 14.3% Mixed = 13% Asians = 3.5%.
Tanzania	(Eddy et al. 2007)	No	CS	(*N* = 214) Age: 13–30 years Sex: female Ethnicity: 45% Mchagga 25% Mpare Social Economic Status: 42% students 31.6% farners 13% in business 3.7% teachers 1.7% unemployed 8% nonformal sector work	SCID, EDI	Point prevalence: in the past 12 months AN = 1.9%, BN = 0.5%, OSFED = 4.7%.
Tanzania	(Lugata et al. 2021)	Yes	CS	University students (*N* = 1047) Age: *M* = 24.2 years (SD = 7) Sex: females and males Ethnicity: NR Socio‐economic status: NR	SCOFF	Scored above cutoff SCOFF (≥ 2): 7.4%.
Uganda	(Abaatyo et al. 2025)	Yes	CS	Medical students (BA) (*N* = 224) Age 27 (SD = 5.6) Sex: 37% females, 73% males Ethnicity: NR Socio‐economic status: 70% was employed during study. Study program is at private university.	SCOFF	Scored above cutoff SCOFF (≥ 2): 16.5% total sample. Scored above cutoff SCOFF (≥ 2): Males: 14.2%. Females: 46.0% Scored above cutoff SCOFF bulimia subscale: 13.4% (total sample)
Uganda	(Kinyanda et al. 2013)	Yes	CS	Females and males (*N* = 1587) Aged 3–19 years Sex: females and males Ethnicity: NR Social Economic Status: family income (UGX): 11.6% < 15,000 7% 15,000‐99,000 2.5% > 100,000	MINI	Point prevalence in the past 3 months ED = 0.7%.
Zimbabwe	(Buchan and Gregory 1984)	Yes	CR	(*N* = 1) Age: 20 years old Sex: female Ethnicity: African Socio‐economic status: NR	EAT‐40	Case presentation of a 20 years old women with a diagnosis of AN lived in the United Kingdom age 2–6 years old. Through scholarships she attended boarding schools in Zimbabwe and university in the United Kingdom EAT score = 78. Weight during admission at the age of 22 was 42 kg (Height unknown but 50 kg was suggested as optimal weight).
Zimbabwe	(Hooper and Garner 1986)	Yes	CS	High school students (*N* = 399) Age: NR Sex: female Ethnicity: NR Social Economic Status: NR	EDI	Score above the clinical cutoff = 10.4%.
Studies on prevalence on symptoms of ED pathology and behaviors (e.g., dieting, body image)						
South Africa	(Mchiza 2014)	Yes	CS	Adolescents (*N* = 220) Age: 10–14 years Sex: female Ethnicity: NR Socio‐economic status: NR	National health survey	The majority had a negative body image. 68% reported a distorted body image. 17% reported using weight loss behaviors (e.g., food restriction and extensive exercising).
Tanzania	(Eddy et al. 2007)	No	CS	(*N* = 214) Aged 13–30 years 45% Mchagga 25% Mpare Social Economic Status: Economic Status: 42% students 31.6% farmers 13% in business 3.7% teachers 1.7% unemployed 8% nonformal sector work	SCID‐IV‐I/P, EDI	Binge eating: at least episode during the last month = 9.8%. Self‐induced vomiting: at least once during the last month = 5%. ED symptoms evaluated by the SCID such as fear of weight gain, body image disturbance, overvaluation of weight/shape = 39.8%. Specifically, fear of gaining weight = 20.9%, body image disturbance = 20.9%, overvaluation of weight/shape = 14.7%. Mean BMI 22.5 (SD = 3.4; range 15.1–35.1).

Abbreviations: AN, anorexia nervosa; BIQ, body image questionnaire; BITE, bulimic investigatory test, Edinburgh; BMI, body mass index (weight/kg^2^); BN, bulimia nervosa; BSQ, body shape questionnaire; CR, case‐report; CS, cross‐sectional; EAT, eating attitude test; ED, eating disorder; EDI, eating disorder inventory; MINI, mini‐international neuropsychiatric interview; NR, not reported; PDSQ, psychiatric diagnostic screening questionnaire; SCID, structured clinical interview for DSM; SCOFF, sick, control, one stone, fat, food; VS, validation study; WCS, weight concerns scale.

### 3.2 Prevalence

An important consideration when evaluating the methodologies of the included studies in the present review is that all recruited participants were high school or university students. This suggests that the samples were likely drawn from urban rather than rural settings, contexts generally associated with higher ED prevalence and increased severity of pathology (AlHadi et al. 2022). In addition, mainly female individuals were included in the samples, who are more prone to EDs (Culbert et al. 2021). All of the studies reporting on clinical cutoffs used self‐report measures rather than clinical interviews by trained ED professionals and allow room for cultural interpretation of the questions. Moreover, most of the (self‐report) assessment measures were not validated or reported limited psychometric information. Therefore, the present Research Forum was not able to reliably interpret and provide specific explanations for the findings based on self‐reports.

### 3.3 Prevalence of Eating Disorders

Overall, data on ED prevalences across Southern Africa are severely limited (see Table 1). Of note, most of the studies used DSM‐IV (APA 1994) or older criteria. Therefore, it is likely that some individuals (0.2%: 4 individuals out of 1476 participants in all included papers in van Hoeken et al. 2016) in these African studies would have fulfilled AN criteria when applying the DSM‐5 criteria (APA 2022). Although the first identification of AN in Southern Africa was reported in 1979 in a 20‐year‐old female in Zimbabwe (Buchan and Gregory 1984), only two quantitative studies subsequently examined the point prevalence of AN in this region. Furthermore, although no restrictions on age were implemented in the selection process, all included study samples in the present Research Forum reported on female individuals aged 13–30.

#### 3.3.1 Madagascar

One study examined pica in a large sample of Malagasy individuals aged five and older. A higher prevalence (between 18% and 85.2%) of amylophagia (the consumption of starch), geophagy, and several other pica‐related behaviors (no associations with pregnancy) was found (Golden et al. 2012). However, an excessive desire to consume starch and carbohydrate‐rich foods may be explained by Madagascar's status as one of the poorest countries in the world (WorldBank 2024), where the local diet consists of primarily rice and starchy dishes. This diet, typical of impoverished countries, is associated with iron deficiencies, which in turn have been associated with pica behaviors, particularly the chewing of cornstarch (gum) throughout the day (Maksoud et al. 2010). Interestingly, high levels of amylophagia and geophagy were reported in female individuals of African ethnicity in other parts of the world (Grigsby 2013). This interpretation of disordered eating behavior in the context of local diets highlights the importance of acknowledging cultural variations in what may be interpreted as disordered eating behavior versus culturally appropriate or simply necessary (limited food availability) eating behaviors (Levinson and Brosof 2016).

#### 3.3.2 South Africa

A study published in the late 70's reported a 20‐fold increase in AN admissions in South Africa (Norris 1979). Subsequently, among female school students, the prevalence of AN was estimated at 2.9% (Ballot et al. 1981). However, this figure was not based on DSM criteria, but on a combination of measured body dimensions (e.g., weight and height) and self‐reported perception of body image (do you consider yourself to be underweight, overweight, or normal weight?) and eating behaviors. This prevalence therefore reflects individuals who might have met the criteria for “AN‐like” pathology rather than a formal diagnosis.

#### 3.3.3 Tanzania

Based on the SCID, AN point prevalence was estimated at 1.9% (Eddy et al. 2007). In addition, Eddy et al. (2007), were the only researchers to examine the point prevalence of BN and Other Specified Feeding and Eating Disorders (OSFED) in the SADC, reporting an estimated point prevalence of 0.5% for BN and 4.7% for OSFED. The estimated BN prevalence may resonate with the suggestion that BN is a more culture‐bound syndrome (Levinson and Brosof 2016). In this context, eating large amounts of food when it is available might be adaptive rather than pathological.

#### 3.3.4 Uganda

Based on the MINI, ED point prevalence was estimated at 0.7% among female and male individuals aged 3–19 years old (Kinyanda et al. 2013). This relatively low estimate rate is potentially partly attributable to the age distribution of the sample. Nearly half of the participants were aged ≤ 10 years, an age range in which EDs are less common (López‐Gil et al. 2023; Sanzari et al. 2023).

### 3.4 Prevalence of Individuals Scoring Above Clinical Cutoffs on an Eating Disorder Self‐Report

#### 3.4.1 Mozambique

In Mozambique, the WCS was completed. Table 1 shows that 15.7% of the female college students scored above the clinical cutoff (Da Silva et al. 2017).

#### 3.4.2 South Africa

Among female high school students in South Africa, the EAT‐26 was administered. The prevalence was 37.5% among those of African ethnicity and 20.7% among those of Caucasian ethnicity (Szabo and Hollands 1995). Similar studies, where data were collected from both urban and rural areas, reported prevalence estimates of 3.1% in rural areas (Szabo and Allwood 2004a) and 18.7% among those of African ethnicity and 18.6% among those of Caucasian ethnicity in urban areas (Szabo and Allwood 2004b). Among female and male individuals attending high school and university, the prevalence was estimated at 3.5% based on the EAT‐26 (Le Grange et al. 2006) and 9% based on the EAT‐40 (le Grange et al. 1998). Based on self‐report without the use of a validated screener among female and male university students, lifetime prevalence was estimated around 15% among individuals of African and Caucasian origin and 13% and 3.5% among individuals of mixed and Asian origin, respectively (Swartz and Sheward 1995). Similarly, an estimated 2.9% 12‐months prevalence was self‐reported among female high school students (Ballot et al. 1981).

#### 3.4.3 Tanzania

The SCOFF was conducted among female and male university students. A total of 7.4% scored above the clinical cutoff (Lugata et al. 2021).

#### 3.4.4 Uganda

The SCOFF was conducted among medical students. A total of 46.0% of female and 14.2% of male individuals (16.5% of the total sample) scored above the cutoff (Abaatyo et al. 2025).

#### 3.4.5 Zimbabwe

The EDI was administered among high school students. A total of 10.4% scored above the clinical cutoff (Hooper and Garner 1986).

### 3.5 Prevalence of Eating Disorder Symptoms

Very few studies examined the prevalence of ED symptoms/behaviors, all assessed by self‐report, and findings were limited to (adolescent) female individuals in Mozambique, South Africa, and Tanzania.

#### 3.5.1 Mozambique

Weight concerns were examined among female college students. A total of 15.7% reported these concerns (da Silva et al. 2014).

#### 3.5.2 South Africa

Among female adolescents, 17% reported extreme weight‐loss behaviors. Additionally, 68% reported a distorted body image (Mchiza 2014).

#### 3.5.3 Tanzania

The prevalence of self‐induced vomiting in adolescent and young female individuals was 5%; binge eating was 9.8%. Finally, 39.8% reported overvaluation of shape and weight (Eddy et al. 2007).

## 4 Discussion

The aim of the present Research Forum was to review existing literature that estimates the prevalence of EDs, of individuals who score above a clinical cutoff on an ED self‐report assessment tool, and of ED symptoms in Southern Africa (SADC 1980a). Literature was searched in English and in the relevant Southern African languages. No studies were conducted in Comoros, the DRC, Eswatini, Lesotho, Malawi, Mauritius, Namibia, or Zambia, which may stem from limited research infrastructure, funding shortages, and stigma around mental health (Nicholas et al. 2022). Data on the prevalence of EDs and of individuals scoring above a clinical cutoff in Southern Africa were limited but confirm that ED pathology is present in this region. Similarly, whilst there is a lack of data, in available studies on ED symptoms (e.g., binge eating and overvaluation of shape and weight) were reported by 5%–39.8% of individuals in Southern Africa and align with global observations (Hernández et al. 2022; Melisse et al. 2020; Thomas et al. 2016). These findings highlight the importance of attention for EDs in Southern Africa, where EDs are understudied. Based on the findings in the present Research Forum, we propose a number of key recommendations for how to move the field forward in this emerging area of research.

First, when aiming to research and understand EDs in Southern African countries, it is key to do so in the context of globalization and to research if and how globalization processes in Southern Africa are associated with ED prevalence and pathology. Globalization coincides with urbanization, exposure to the western thin ideal, partially presented through media, as well as the introduction of Western types of food, and a more sedentary lifestyle. In other countries across the world these globalization processes are consistently associated with higher ED prevalence and ED pathology (Melisse et al. 2020; Thomas et al. 2016). Recent data indicate that exposure to globalization‐related risk factors is growing across Southern Africa (GSMA 2023). Mobile phone penetration exceeded 80% in many countries and social media usage continues to rise, particularly among adolescents and young adults. In addition, access to internet‐enabled devices increased, even in rural areas (Greenleaf et al. 2025; GSMA 2023; Pew Research Center 2022). Despite many regions in Southern Africa going through globalization at a rather rapid pace, only one of the studies included in this Research Forum reported on associations between globalization processes and ED pathology (Eddy et al. 2007). We suggest that future studies researching ED prevalence and pathology in Southern Africa should focus on three broad globalization processes. First: urbanization, more specifically, the differences between urban and rural areas. Global research suggests higher levels of ED pathology in urban areas (Keski‐Rahkonen et al. 2018). The majority of studies included in the present Research Forum involved university or high school students, samples that typically represented more urbanized areas (as well as higher socio‐economic status). Yet, there was some evidence of higher levels of ED pathology in urban, compared to rural areas (Eddy et al. 2007; Malete et al. 2013; Prioreschi et al. 2017; Senekal et al. 2001; Szabo and Allwood 2004a, 2004b; Wassenaar et al. 2000). Overall, studies in rural areas are scarce, and future studies should aim to include a broad reflection of society, including participants from rural areas. Additionally, urban (versus rural) origin was commonly based on participants' geographic background (e.g., farms/small villages vs. towns/cities), but future research may need to include other measures of urbanization (e.g., housing situations, access to food shops, access to internet).

Second: shifts in beauty ideals. Globalization is associated with exposure to Western beauty ideals, for example through access to Western social media, particularly the slimmer body. This in turn has been hypothesized to be associated with sociocultural shifts in appearance norms, from curvy to thin body preferences (Gerbasi et al. 2014; Gordon 2001; Nasser et al. 2001). It is important to examine if and how beauty ideals may be changing in Southern Africa, as this thin‐body ideal internalization is associated with body dissatisfaction and consequently EDs (Stice et al. 2010). Body dissatisfaction is globally one of the important risk factors for ED pathology (Melisse et al. 2024). Potentially thus, globalization processes such as westernization, the associated changes in beauty ideals and novel social norms for a thinner body reinforce disordered eating attitudes and behaviors (Gerbasi et al. 2014). Interestingly, a recent study in Kenya (a country neighboring Tanzania and Uganda and sharing a similar colonial past and cultural aspects, e.g., Swahili language) found that globalization was associated with changed expectations for appearance, from a curvy to a thinner body (Mutiso et al. 2022). In line with the recently increased globalization, a handful of more recent studies examining body image in Southern African countries report high prevalence estimates of body dissatisfaction, particularly those in urban areas (Eddy et al. 2007; Prioreschi et al. 2017). In Botswana, adolescents with a BMI of > 25 and > 30 reported greater dissatisfaction with their weight and body proportions compared to peers of optimal weight (Malete et al. 2013). Similar results were found in South Africa, where young female participants at the extreme lower ends of the BMI index range report a distorted body image, drive for thinness, and high body dissatisfaction (Mchiza 2014). In addition, a large proportion of adolescents were struggling with body image (83.5%) and even when underweight desiring “underweight” body shapes (5.8%; Pedro et al. 2016). Overall, these data hint at a move away from the traditional fuller beauty ideal towards a thinner beauty ideal in these regions. Conversely, only in one study South African participants reported the desire to be overweight (Pedro et al. 2016), highlighting the complex interaction between cultural values and globalization (Melisse, El Khazen, and de El Khazen 2025). Taken together future studies should examine associations between globalization (e.g., access to Western social media), body images issues (e.g., thin‐ideal internalization and body dissatisfaction) and ED prevalence and pathology.

Third: access to Western food. The shift towards globalization often coincided with a more sedentary lifestyle, and an increase in the availability of processed (fast) food (Gorrell et al. 2019; Melisse, Fakhri, et al. 2025; Pike et al. 2014; Swain 2006), both associated with a high BMI (Eladawi et al. 2018). In both Western and non‐Western samples, BMI was positively associated with ED pathology (Melisse et al. 2022, 2020). Indeed, in both Botswana and South Africa associations were found between high BMI and ED pathology, highlighting the importance of including these factors into future ED studies examining ED pathology in Southern African countries.

A second line of research that requires attention is the association between food insecurity and ED pathology. As reported in Western samples, including the Minnesota starvation study, food availability, and uncertainty about its availability may in fact be associated with ED pathology. Food scarcity may starve the brain (Keys 1950) and consequently, ED cognitions may occur, as well as behaviors like binge eating during the availability of food (Keys 1950; Olson et al. 2007). Subsequently, binge‐eating behaviors may be an adaptive response to food scarcity and should be interpreted as such when reported in diagnostic interviews or questionnaires. In addition, malnourishment may also be associated with eating non‐edible products as perceived in pica (Miao et al. 2015). Somewhat surprisingly, none of the included studies examined associations between food insecurity and ED pathology or even reported on (perceived or expected) food availability or shortages. Yet, all the Southern African countries faced food shortages, or at least food unpredictability (i.e., food abundance one year, food shortage the next, usually determined by drought or excessive rains) in the last few decades (WorldBank 2024). When examining ED prevalence and pathology, researchers should consider the interplay between both globalization and westernized body ideals on the one hand and food insecurity on the other. For example, it is possible that the student samples included in the papers included in the present Research Forum represented a new “middle class.” They might be caught in a quandary where they are negatively impacted by globalization, which generally promotes the Western thin ideal, while also experiencing food insecurity, therefore implicating them in multiple ways in the development of binge‐eating behaviors and other ED pathology (Stice et al. 2010). Moreover, it is likely that the high prevalence of pica behaviors in Madagascar (Golden et al. 2012) was attributable to the low nutritional local diet, which is a direct result of food shortages. Future ED research in Southern African countries should consider food availability, and how food insecurity may result in pica behaviors or a starved brain, which in turn may contribute to ED cognitions and behaviors (Keys 1950; Olson et al. 2007).

A third area in need of investigation is whether and how race and ethnicity may be associated with the prevalence of EDs and symptom presentation, bearing in mind that such differences are more likely attributable to underlying cultural, social, and environmental factors than to ethnicity itself. For the purposes of this Research Forum we use the terms race and ethnicity, yet we are aware of the complexity of this construct and its definitions being founded from Western perspectives. Differences between ethnic groups may be associated with cultural norms, levels of stigma, socioeconomic environment, and exposure to evolving social pressures, rather than inherent ethnic differences (Cini et al. 2024; Miller and Pumariega 2001; Szabo 2019). Literature shows, for example, that whilst there are higher rates of BN in Latino, Asian, and African American female and male individuals relative to non‐Latin Caucasian individuals, rates of AN and BED do not differ (Marques et al. 2011), and that symptomatology was associated with migration or expatriate status and ethnicity (Abdollahi and Mann 2001; Al Adawi et al. 2002; Salant and Lauderdale 2003; Toselli et al. 2016). It was also suggested that ethnic differences in ED presentation may have implications when examining risk factors (Wildes and Forbush 2015). Data on ethnicity, race, and EDs in Southern Africa is very limited; yet, while conclusions are hard to draw, there are a couple of studies that suggest the importance of continuing this line of research. Namely, two studies reported that ethnicity was not associated with ED pathology (Edwards et al. 2003; le Grange et al. 1998). However, other studies found that individuals of African ethnicity reported higher levels of ED pathology compared to Caucasian, mixed, or Asian samples (le Grange et al. 1998; Swartz and Sheward 1995; Szabo and Hollands 1995; Wassenaar et al. 2000). Conversely, one study found that Caucasian ethnicity, and not African ethnicity, was associated with body image distortions (Senekal et al. 2001). Differences in symptom presentation were also observed. For instance, African ethnicity was associated with a drive for thinness and paradoxically, a higher BMI, while Caucasian participants reported higher levels of body dissatisfaction (Wassenaar et al. 2000). Additionally, in Zimbabwe, mixed ethnicity was associated with binge‐eating behaviors, while both Caucasian and mixed ethnicity were associated with restrictive eating behaviors (Hooper and Garner 1986). A South African study including school students aged 4–12 found that associations between ethnicity (African versus Caucasian) and ED behaviors differed across the developmental stages, but without a clear trend (Mould et al. 2011). Of note, whilst Asian samples were only included in three studies, all from South Africa, evidence suggested lower ED pathology in those with Asian ethnicity (le Grange et al. 1998; Swartz and Sheward 1995; Wassenaar et al. 2000). Of note, this does not reflect epidemiological ED studies in Asia (Alfalahi et al. 2022; Thomas et al. 2016). Possibly, the immigrant Asian community in South Africa represented a distinct cultural group, and may not be comparable to samples in Asia. In conclusion, future research should examine if ethnicity indeed is associated with the prevalence and presentation of EDs, or if ethnic identity may act as a proxy for the interplay between social, cultural, economic and structural exposures including stigma, changing beauty norms and access to care, which is associated with risk for an ED and symptom expression across groups (Cheng et al. 2019; Cini et al. 2024; Miller and Pumariega 2001; Szabo 2019). This should be done across various cultural contexts and age groups. This approach clarifies how cultural, social, and structural factors are associated with group differences in EDs, and prevents attributing risk or ED prevalence to ethnicity alone. For the purposes of this Research Forum the terms race and ethnicity were used; yet, the complexity of this construct should be noted, given its association to (colonial) history and their place in current politics. Moreover, definitions of race and ethnicity were founded from Western perspectives. Future research should therefore aim to include factors such as cultural habits and beliefs, (colonial) history, social economic status, urbanization, education, language, etcetera.

A fourth research gap that urgently requires future attention is the role of sex/gender in Southern Africa. Global research firmly acknowledges gender differences in the prevalence of EDs, with higher prevalences in female individuals across all EDs relative to male individuals (Hay et al. 2023; Qian et al. 2021; Silén and Keski‐Rahkonen 2022). The majority of studies in the current Research Forum examining prevalences include mainly young female samples (Mchiza 2014; Mould et al. 2011; Szabo and Hollands 1995; Wassenaar et al. 2000). Only three studies included both female and male participants (Edwards et al. 2003; le Grange et al. 1998; Swartz and Sheward 1995). While Swartz and Sheward (1995) reported similar prevalences of ED pathology across sex, the study by le Grange et al. (1998) found that female individuals reported more severe ED pathology compared to male individuals. Additionally, in Tanzania, Eddy et al. (2007) found high prevalences of ED cognitions (nearly 40%) and behaviors such as binge eating and self‐induced vomiting in young Tanzanian female individuals. These data were comparable to Western and non‐Western studies recognizing higher prevalence of ED pathology in female individuals compared to male individuals (Nagl et al. 2016; van Eeden et al. 2023). As the role of sex remains unclear, studies that further investigate sex and gender‐related ED prevalences in Southern African countries are needed, as this may, for example, help to identify potential sex or gender differences in clinical presentation and as such may contribute to an earlier diagnosis.

Beyond sex, individuals whose gender identity does not align with their sex assigned at birth (e.g., transgender or nonbinary) seemed to experience high levels of ED symptomatology compared to cisgender individuals (Breton et al. 2023; Rasmussen et al. 2025). Although this is potentially associated with developing EDs (Rasmussen et al. 2025), prevalences in nonbinary and transgender individuals were not specifically examined in any Southern African study as of yet. This fourth research gap highlights the importance of incorporating sex and gender diversity when addressing EDs across the southern African region.

Fifth, to obtain an accurate estimate of ED prevalence, it is important to acknowledge potential cultural variations in what is considered disordered eating versus culturally appropriate eating behavior. For example, understanding eating large amounts of starch as the local diet rather than an ED behavior (Golden et al. 2012). But also considering cultural variations in food habits and routines, as well as the cultural meaning of food and food rituals. For example, in some Southern African communities, eating large portions may be culturally valued and not indicative of binge‐eating behavior as defined by Western diagnostic criteria (Knettel et al. 2018). This highlights the importance of culturally sensitive diagnostic frameworks that take into account local norms and meanings around food. Future work should examine how local food practices and cultural meanings relate to the perception of disordered eating to determine whether adaptations of existing diagnostic frameworks are warranted.

A sixth and essential priority for future work involves the validation of existing diagnostic instruments. Studies should examine whether these are culturally and contextually appropriate for diverse populations in Southern African countries. Some papers included in the present Research Forum used assessment tools, which were not validated for the culture at hand, risking inaccurate diagnoses and a distorted understanding of EDs in this region (Marques et al. 2011; Udo and Grilo 2018). Potentially, these assessment tools need to be culturally adapted, as Western instruments might fail to capture local meanings of food and eating related distress. For example, in Zulu communities, BN was not referred to as ‘ibhulimia’ but rather to ‘isifosokudla’ (the illness of food), ‘ukuminza’ (to gulp down), and ‘isifo sokuhlanzaukudla’ (the illness of bringing up food; van Huyssteen 2005). This suggests that knowledge of local terminology is key when conducting ED research in Southern African countries and that researchers should also be aware of alternative English terms potentially used in the region, such as “food habits” “eating distress” or “nutritional problems” (Abaatyo et al. 2024; Pedro et al. 2016), which may not be captured by the conventional search terms in the present Research Forum. Linguistic variations indicate that the use of Western terminology may obscure locally expressed forms of ED pathology. Therefore, for the present Research Forum, literature was searched in English and in the relevant Southern African languages, attempting to capture region‐specific terminology. Future work should, however, include identification of local idioms and collaboration with local experts to refine search strategies and assessment tools. Furthermore, collaborations between experienced international ED clinicians and researchers and local experts will be important in assessing whether the Western standard ED assessment tools are in fact appropriate for Southern African samples. If not, future research should consider the development and validation of culturally sensitive ED assessment tools that account for local and cultural eating behaviors (e.g., eating large amounts of food when it is available) and local diets as well as variations in the understanding and terminology surrounding EDs, and regional differences in body weight norms, such as the higher prevalence of underweight individuals in Southern Africa (11.5% vs. < 5% in Western countries), which could contribute to the under‐recognition of disorders like AN (Fallatah et al. 2015; WHO 2021).

The lack of validated assessment tools is associated with lower awareness among health‐care providers and the general population. Identifying instruments that are valid and reliable locally can increase ED awareness, lower stigma, improve detection, and potentially inform the development of tailored intervention strategies in the region (Marques et al. 2011; Udo and Grilo 2018). Poignantly, in a study among mental health‐care providers in Tanzania, a special education teacher explained why mental health‐care providers do not observe EDs: ‘I am not aware of any AN, BN, or other EDs. Most Tanzanians eat food when it is available, in as large quantities as possible’ (Knettel et al. 2018). Of note, underreporting of BED, being the most common ED in other parts of the world (AlHadi et al. 2022; Keski‐Rahkonen and Mustelin 2016; Thomas et al. 2016), is likely also due to its formal DSM‐5 recognition in 2013. In all included 14 studies, 16 researchers were local researchers, and when these local experts collaborate with international ED professionals, awareness can be raised among health care professionals; in public health surveillance efforts, educational institutions and community‐based interventions can be developed. Worryingly, owing to the absence of culturally sensitive ED assessment tools, the lack of access to care (van Hoeken et al. 2016; WHO 2014), and the lack of specialized therapists (Philip et al. 2023), most Southern African individuals with EDs may never receive a formal diagnosis or specialized care (van Hoeken et al. 2016).

The present Research Forum has several limitations. Firstly, there is a serious scarcity of data. It is important to note that the majority of studies included in the present Research Forum are relatively old, with many dating back to the 1980s and 1990s (Hooper and Garner 1986; Szabo and Hollands 1995), and only a few being more recent (Abaatyo et al. 2025; Lugata et al. 2021). The age of these studies may limit the relevance and generalizability of their findings, as social and cultural attitudes towards EDs and ethnicity may have changed over time. This is particularly pertinent for South Africa, whose unique cultural history and status as a member of the SADC may have resulted in an earlier and more pronounced trajectory towards globalization compared to other countries in the region (SADC 2024). In addition, studies vary largely in terms of location, highlighting large cultural variations among study findings and so limiting the generalizability of findings. Due to the limited and heterogeneous nature of research on EDs and disordered eating behaviors in Southern Africa, a combined search strategy was applied to maximize sensitivity. The distinction between clinical and non‐clinical populations, as well as formal diagnostic criteria, was often unclear or inconsistently reported across studies. A unified approach allowed for comprehensive identification of relevant literature, with categorization by population type, methodology, and outcomes conducted during screening and data extraction.

Study samples were homogenous and included mainly female individuals, and mostly those of young age. This means that results should be interpreted with caution. Namely, ED prevalence is higher in females, and particularly young individuals (Hay et al. 2023; van Eeden et al. 2023). This may have further biased findings. Moreover, findings may not be transferable to individuals identifying with other sexes. Importantly, only the terms female or male individuals were used in the included papers. It is therefore unknown whether the studies included cisgender women only or applied a more inclusive definition of “female individuals”. Furthermore, most of the data came from young populations from developed regions, and as such, findings should be interpreted within the context of (relatively) higher socio‐economic status and levels of education. It may well be that this same study design, including older (male) populations from more rural areas with lower socio‐economic status, would result in entirely different outcomes. Future research should also examine other potential correlates not covered in the present Research Forum, including educational attainment and literacy (Afrobarometer 2023), as well as sociopolitical or environmental conditions such as climate‐related displacement (UNESCO 2023; WMO 2023), all of which may be associated with EDs in Southern Africa (Mchiza 2014; Rodgers et al. 2023). Finally, usage of various diagnostic interviews and self‐report assessments, which were potentially insensitive, challenged comparisons between regions and with existing literature.

### 4.1 Clinical Recommendations and Implications

Globalization could help improve accessibility to formal health care through the implementation of eHealth or paper‐pencil self‐help programs such as guided self‐help versions of CBT for ED and CBT‐E (Fairburn 2008). Such programs are (cost‐)effective in Western countries (Melisse, Blankers, et al. 2023; Melisse, den van Berg, and De Beurs 2023). It would be beneficial to assess the effectiveness of such programs in regions of Southern Africa, where accessibility to care is limited. Furthermore, preventative programs offering psycho‐education may decrease the stigma of psychotherapy and increase treatment‐seeking behavior (Honein‐AbouHaidar et al. 2016; Williams et al. 2013). In addition, training for primary care providers might improve early detection and referrals for treatment (Philip et al. 2023; van Hoeken et al. 2016). Moreover, globalization is associated with reduced traditional social support, yet seeing that participating in social support groups was associated with reduced EDs (Van Zyl et al. 2012), these traditional social support structures should be boosted.

## 5 Conclusion

In sum, findings from the present Research Forum underlined a research gap and therefore the importance of addressing EDs in Southern Africa. We highly recommend future work to focus on understanding the interplay between globalization and food insecurity and the presentation of ED symptomatology, whilst considering the role of ethnicity, sex, and gender. Future studies should evaluate the appropriateness of (Western) standardized ED assessment tools and include more diverse samples. We recommend that increasing awareness of EDs, running future studies, and developing interventions occurs through close collaboration between international ED experts and local researchers and clinicians.

## Author Contributions

**Bernou Melisse:** conceptualization, investigation, writing – original draft, methodology, project administration, resources, data curation. **Jojanneke M. Bijsterbosch:** resources, writing – review and editing. **Lot Sternheim:** resources, writing – original draft, methodology.

## Funding

The authors have nothing to report.

## Disclosure

AI tools were not used.

## Ethics Statement

The current study was considered exempt from ethical approval.

## Consent

The authors have nothing to report.

## Conflicts of Interest

The authors declare no conflicts of interest.

## Data Availability

Data sharing not applicable to this article as no datasets were generated or analysed during the current study.

## Appendix A

*Search terms and phrases in Southern African languages used to identify relevant studies*.

(“Eating disorders” OR “Anorexia Nervosa” OR “Bulimia Nervosa” OR “Binge Eating Disorder” OR “Disturbed Eating Behavior” OR “Eating Disorder Behavior” OR “Eating Attitudes”) AND (“Southern Africa” OR “Angola” OR “Botswana” OR “Comoros” OR “the Democratic republic of Congo (DRC)” OR “Eswatini” OR “Lesotho” OR “Madagascar” OR “Malawi” OR “Mauritius” OR “Mozambique” OR “Namibia” OR “Seychelles” OR “South Africa” OR “United Republic of Tanzania” OR “Zambia” OR “Zimbabwe”)

## Appendix B

*English search terms and phrases used to identify relevant studies*.

Afrikaans: eetstoornis

English: eating disorder

Ndebele: isifo sokudla

Sepedi: bothata bja go ja

Sesotho: bothata ba ho ja

Setswana: bothata jwa dijo

SiSwati: ngumlamo wokudla

Swahili: tatizo la kula

Tshivenda: vhulwadze ha u kondelela

Xhosa: ingxaki yokutya

Zulu: isifo sokudla

Xitsonga: xihlamariso xa swi dyuharhu

## Appendix C

*NEWCASTLE – OTTAWA QUALITY ASSESSMENT SCALE* (*adapted for cross sectional studies*) (Modesti et al. 2016).

### Selection: (Maximum 5 Stars)

1. Representativeness of the sample:
  - Truly representative of the average in the target population. * (all subjects or random sampling)
  - Somewhat representative of the average in the target population. * (nonrandom sampling)
  - Selected group of users.
  - No description of the sampling strategy.
2. Sample size:
  - Justified and satisfactory. *
  - Not justified.
3. Non‐respondents:
  - Comparability between respondents and non‐respondents' characteristics is established, and the response rate is satisfactory. *
  - The response rate is unsatisfactory, or the comparability between respondents and non‐respondents is unsatisfactory.
  - No description of the response rate or the characteristics of the responders and the non‐responders.
4. Ascertainment of the exposure (risk factor):
  - Validated measurement tool. **
  - Non‐validated measurement tool, but the tool is available or described.*
  - No description of the measurement tool.

### Comparability: Maximum 2 Stars)

1. The subjects in different outcome groups are comparable, based on the study design or analysis. Confounding factors are controlled.
  - The study controls for the most important factor (select one). *
  - The study controls for any additional factor. *

### Outcome: (Maximum 3 Stars)

1. Assessment of the outcome:
  - Independent blind assessment. **
  - Record linkage. **
  - Self‐report. *
  - No description.
2. Statistical test:
  - The statistical test used to analyze the data is clearly described and appropriate, and the measurement of the association is presented, including confidence intervals and the probability level (*p* value). *
  - The statistical test is not appropriate, not described, or incomplete.
    This scale has been adapted from the Newcastle‐Ottawa Quality Assessment Scale for cohort studies to perform a quality assessment of cross‐sectional studies for the systematic review, “Eating Disorders in the Arab world: a literature review”.
